# Health policy and systems research in access to medicines: a prioritized agenda for low- and middle-income countries

**DOI:** 10.1186/1478-4505-11-37

**Published:** 2013-10-14

**Authors:** Maryam Bigdeli, Dena Javadi, Joelle Hoebert, Richard Laing, Kent Ranson

**Affiliations:** 1 Alliance for Health Policy and Systems Research, World Health Organization, 20, avenue Appia, 1211 Geneva, Switzerland; 2 Utrecht Institute for Pharmaceutical Sciences, Division of Pharmacoepidemiology and Clinical Pharmacology, Utrecht University, P.O. Box 80 082, 3508 TB Utrecht, The Netherlands; 3 Department of Essential Medicines and Health Products, World Health Organization, 20, avenue Appia, 1211 Geneva, Switzerland

**Keywords:** Access to medicines, Health systems, Health systems research, Priority setting

## Abstract

**Objectives:**

To identify priority policy issues in access to medicines (ATM) relevant for low- and middle-income countries, to identify research questions that would help address these policy issues, and to prioritize these research questions in a health policy and systems research (HPSR) agenda.

**Methods:**

The study involved i) country- and regional-level priority-setting exercises performed in 17 countries across five regions, with a desk review of relevant grey and published literature combined with mapping and interviews of national and regional stakeholders; ii) interviews with global-level stakeholders; iii) a scoping of published literature; and iv) a consensus building exercise with global stakeholders which resulted in the formulation and ranking of HPSR questions in the field of ATM.

**Results:**

A list of 18 priority policy issues was established following analysis of country-, regional-, and global-level exercises. Eighteen research questions were formulated during the global stakeholders’ meeting and ranked according to four ranking criteria (innovation, impact on health and health systems, equity, and lack of research). The top three research questions were: i) In risk protection schemes, which innovations and policies improve equitable access to and appropriate use of medicines, sustainability of the insurance system, and financial impact on the insured? ii) How can stakeholders use the information available in the system, e.g., price, availability, quality, utilization, registration, procurement, in a transparent way towards improving access and use of medicines? and iii) How do policies and other interventions into private markets, such as information, subsidies, price controls, donation, regulatory mechanisms, promotion practices, etc., impact on access to and appropriate use of medicines?

**Conclusions:**

Our HPSR agenda adopts a health systems perspective and will guide relevant, innovative research, likely to bear an impact on health, health systems and equity.

## Background

The provision of reliable access to affordable, quality medicines is a necessary component of functioning health systems [1]. However, access to and use of essential medicines^a^ is often poor in low- and middle-income countries (LMICs), with particular challenges faced by the poor. In the formal sector of LMICs, average availability of medicines is 35% in public facilities and 66% in the private sector, although prices are often unaffordable in the latter [2]. Up to 50% of medicines are inappropriately prescribed or dispensed and 50% are used incorrectly by patients [3]. Health-seeking behaviour towards an unregulated private market is widespread [4,5]; substandard and counterfeit medicines are also prominent [6-8]. Medicines typically account for 20% to 60% of health spending [2], and 50% to 90% of this amount is out-of-pocket [9].

There is a need to support Health Policy and Systems Research (HPSR) in the field of medicines in resource poor settings and the topic has been highlighted as an important area of research by the Taskforce on Health Systems Research [10]. A bibliometric survey of publications in HPSR in LMICs showed 648 publications on medicines between 2003 and 2009 [11]. This figure represents only 10% of global publications on medicines and, of these, only 50% are written by authors from LMICs.

Given limited resources available for HPSR in LMICs and the large number of issues to be researched, priority setting is an essential initial step to undertaking meaningful, policy-relevant research. The World Report on Knowledge for Better Health reports that policy-makers, researchers and users are usually unaware of how research priorities are set [12]; HPSR is often driven by donor support rather than by country needs [13,14]. In relation to medicines, priority-setting exercises have usually aimed at defining lists of priority medicines to address global disease burden [15] or specific health problems faced by vulnerable groups such as mothers and children [16], but they have not defined a HPSR agenda. The proceedings of the International Conference on Improving the Use of Medicines present key recommendations for research [17]. However, these are almost 100 research recommendations across 19 themes and are not translated into prioritized research questions. Moreover, as they emanate from the proceedings of a scientific conference, these recommendations reflect gaps in academic work presented, which may not adequately reflect the concerns of decisions-makers or patients and communities.

This paper presents a priority-setting exercise performed by the Alliance for Health Policy and Systems Research (AHPSR) between 2010 and 2012, with the objectives of: i) identifying priority policy issues in access to medicines (ATM) relevant for LMICs; ii) identifying research questions that would help address these policy issues; and iii) prioritizing these research questions in an actionable HPSR agenda.

## Methods

### Analytical framework

The WHO framework “*Equitable Access to Essential Medicines: A Framework for Collective Action*” [18] was used as a guide. This framework presents four dimensions of ATM: rational selection and use, affordable prices, sustainable financing, and reliable health and supply systems. Each of these four dimensions is influenced by determinants of ATM rooted in local, national and international context [19].

### Stepwise approach

**Figure 1:**
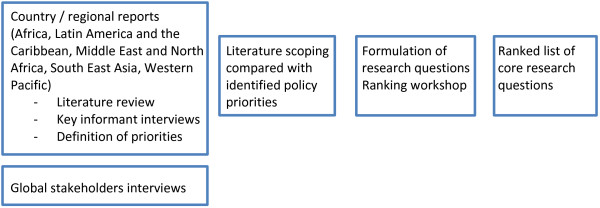
Stepwise approach to priority-setting for access to medicines.

The stepwise approach used to determine research priorities was adapted from Ranson et al. [20,21] and is summarised in Figure 1. First, in 17 countries across five regions, research teams performed an exhaustive literature review, focusing on ATM in their countries and regions, and which covered published and grey literature in local as well as international databases. This was followed by key informant interviews (KIIs) and definition of priority policy issues. This resulted in a series of country and regional reports, which were supplemented by KIIs with global stakeholders. Second, a literature scoping review was conducted to identify existing published research, which was then mapped against the priorities identified at country-, regional- and global-level. The findings of the first two steps were shared with a group of experts during a consultative workshop aimed at formulating priority research questions and ranking these in a prioritized HPSR agenda.

### Country-, regional- and global-level priority setting

Seventeen countries were selected by the AHPSR based on the following criteria: i) the capacity of in-country researchers and institutions; ii) their ability to approach a diverse group of stakeholders in a meaningful dialogue; and iii) their ability to adopt a health system perspective on ATM. Efforts were made to keep a balance between regions and to include both low- and middle-income countries. The analytical framework [18] was presented and discussed in detail with research teams at an inception meeting. Meeting participants also discussed how best to design and implement data collection tools that would capture all elements of the framework and determinants of access at all levels of the health system. Country teams were given general directions for steps to follow in collecting and analysing data. They were asked to: i) perform a search of grey and published literature related to their country and region, and identify existing information on priority policy issues and existing research in ATM; ii) map relevant stakeholders from various groups: researchers, policy makers, health service providers, civil society and NGOs including patient representatives, donor agencies, pharmaceutical companies and other relevant stakeholders; iii) select a sample of stakeholders with adequate representation of all groups; perform KIIs to identify priority policy issues and research questions; iv) derive from these priority policy issues on ATM and provide a list of research questions if possible. Each team conceived search and survey methods adapted to their contexts and in local languages. Table 1 presents the list of countries by region, the number of informants in each country and whether identification of priority policy issues was based on consensus meeting vs. ranking (either in ranking meetings or as a component of KIIs).

**Table 1:** Country-/regional-level approaches to priority setting

**Region**	**Country**	**No. informants**	**Method used to define priority policy issues**
Latin America	Dominican Republic	12	Ranking during face-to-face interviews or web-based surveys
	Suriname	12	
	El Salvador	12	
	Colombia	12	
Africa	Cameroon	102	Consensus meeting validating analysis of KII
	Congo	110	
	Gabon	80	
	Chad	110	
	Rwanda	28	
	Ghana	16	
South East Asia	India	26	Qualitative analysis of KIIs
Western Pacific	Cambodia	NA*	NA*
	Lao PDR	18	Ranking during face-to-face interviews
	Vietnam	21	Ranking during face-to-face interviews
Eastern Mediterranean	Iran	20	Ranking meeting
	Pakistan	21	Consensus meeting validating analysis of KII
	Lebanon	29	Ranking meeting

*The research team in Cambodia did not obtain authorization to conduct key informant interviews. Cambodia results are limited to desk review of published and grey literature.

Institutions were requested to seek ethical approval in their own country or region. Data collection took place between November 2010 and December 2011 and country/regional reports were submitted to the AHPSR between May and December 2011^b^. The reports were read by two reviewers independently (MB and JH). Priority policy issues for each country were extracted and categorized according to the analytical framework. Results of independent data extraction were compared; discrepancies were discussed and resolved by returning to the original report. Cross-cutting priority policy issues appearing in at least three regions were identified and considered as being of high priority. Priority policy issues appearing in fewer than three regions were not considered as high priority.

During the data extraction process, some amendments were made to the original framework. The team observed that there was considerable overlap between issues related to domains “affordable prices” and “sustainable financing”. That is, when issues of affordability as a demand-side aspect were studied, naturally the supply-side, sustainable financing and implications, were also studied and vice versa. Thus, the two domains were merged into one, inclusive of both demand- and supply-side financing. Further, the domain “reliable health and supply system” covered a large number of policy issues and was therefore expanded and sub-domains defined. The final domains used were as follows:

1. Medicine selection and use

2. Sustainable financing and affordable prices of medicines

3. Leadership and governance (reliable health and supply systems)

4. Availability of medicines (reliable health and supply systems)

5. Human resources for health (reliable health and supply systems)

6. Quality of medicines and quality assurance systems (reliable health and supply systems)

7. Medicines information and information systems (reliable health and supply systems)

To collect data on priority policy issues from a global perspective, 23 stakeholders from headquarters and regional offices, of international multilateral and bilateral agencies, academia and NGOs were interviewed. These informants were selected for their expertise and knowledge of ATM issues at international level. Priority policy issues identified at country- and regional-level^c^ were used to design a semi-structured telephone interview guide. In relation to each of the seven domains of the revised framework and through a series of open-ended questions, informants were asked whether they agreed with priority issues defined at country- and regional-level, and whether any issue was missing and would deserve to be prioritized. Interviews were performed between June and November 2011 by researchers external to the AHPSR with 23 informants who responded in their professional capacity. Interviews were conducted in English following email agreement and oral consent at the start of the interview. Interviews were not recorded but transcribed; transcripts were sent back to the interviewees for final agreement. Transcripts were read by two independent readers (JH, who performed the interviews and DJ, who did not) and priority policy issues identified by global key informants were extracted in an Excel spread sheet, according to the modified analytical framework. Results of independent reviews were compared and discrepancies were discussed and resolved by returning to the original transcripts.

Policy issues were categorized according to the seven domains above. Topics were ordered firstly according to the number of regions in which the issue was raised and second according to the number of interviewees who raised the issue at global level.

### Literature scoping

Using the list of priorities identified through the KIIs and the country reports, a search strategy was devised to extract relevant literature. The search strategy was adapted from a review recently published by the AHPSR [11] and is presented in Additional file 1 and described below. As PubMed is limited in its inclusion of relevant LMIC literature, additional sources were considered. Country and regional teams conducted literature reviews on ATM in their local context, using international and local databases, resulting in a comprehensive reference list for each country and region. To supplement the global search, reference lists provided by country and regional teams were included to strengthen the inclusiveness of the literature scoping (see Additional file 2 for the list of databases searched by country, regional and global research teams). Finally, a report by Gray and Suleman as commissioned by the AHPSR was used as well to further broaden the search [22]. These additional sources covered major databases for published and grey literature and databases specific to LMICs. From these, relevant publications and systematic reviews were included in the scoping exercise.

#### Literature search strategy

The main search theme included three concepts: medicines, LMICs, and access. These were combined with specific search terms related to each of the seven domains of the analytical framework. The global search was limited to PubMed 2003–2012 with additional references being drawn from country literature reviews through local and global databases highlighted in Additional file 2. Published papers were screened by two independent researchers and were included based on whether or not they were relevant to access to medicines, set in LMICs, and relevant to the priority areas. Results focusing on vaccines, research and development, legislative details of intellectual property rights, and pharmacoepidemiology were excluded as they were not outlined as priority ATM concerns at this time. Abstracts of included articles were also reviewed by two independent researchers and the following information was extracted for each publication: country; region; urban vs. rural setting focus; level at which the study was conducted, according to the same levels as previously outlined, namely i) patient, household, community, ii) health service delivery, health service provider, iii) health sector, iv) cross-cutting national policies, and v) international level; public vs. private sector focus; study methods: qualitative, quantitative, mixed, systematic review, other (including reviews and commentaries); whether the study presents impact or results of an intervention aimed at improving access to medicines; and which of the 18 policy issues was addressed in the article. Where studies were focused on more than one setting or more than one of the seven priority domains, this has been outlined.

### Formulation of research questions and ranking

A global stakeholders meeting was organized in Bangkok on 6–8 March 2012. Participants from academia were selected based on their records of research and publications in the field of pharmaceuticals and health systems. Non-academia participants were selected to represent decision makers in the public and private sectors, which included multi-lateral agencies, government agencies, NGOs, pharmaceutical companies, and private foundations. The 20 participants included three who lead country-level exercises and five who were interviewed as part of the global stakeholders’ interviews. Participation was voluntary and based on availability. In preparation for the meeting, participants received summaries of individual country- and regional-level priority setting exercises, a background paper detailing the methods and results of country-, regional- and global-level priority setting as well as methods and results of literature scoping, and a spreadsheet presenting the full list of references including abstracts. During the first half of the meeting, 14 participants from academia reviewed the list of priority policy issues resulting from country-, regional-, and global-level research; and agreed on the categories as well as the wording. Once agreement was reached, they formulated research questions corresponding to each of the ATM policy issues identified in the first two steps. This exclusive time for formulation of research questions was designed to overcome the difficulty of translating policy issues into health policy and systems research questions, which required the expertise of researchers. During the second half of the meeting, non-academic participants joined the group, reviewed the list of policy issues, and drafted questions formulated by academia. Consensus was reached on a final set of 18 unranked research questions.

Participants also debated and reached consensus on four ranking criteria, to which they decided to attribute the same weight:

1. Innovation: Will the research stimulate innovation?

2. Impact: Is the resulting research likely to be effective in strengthening health systems and improving health?

3. Equity: Is the research likely to reduce disparities in health outcomes, perceived as unfair?

4. Lack of research: Is there a lack of research on this topic?

In addition, participants suggested and agreed to add an overall score, irrespective of ranking criteria, that would assess the overall importance of each research question in LMICs and proposed that each participant lists his/her top five priority research questions at the end of the questionnaire. The questionnaire was modified to account for these suggestions.

Participants ranked the 18 research questions through an individual anonymous questionnaire. A five-point Likert scale was used for each ranking criteria where one represented low priority and five represented high priority. Three different methods were used for combining scores across participants:

First, the “total score” involved: i) for each participant and question, summing the four ranking criteria (given the consensus decision to assign equal weight to each criterion); and ii) taking the flat (non-weighted) average of scores for each question across all participants. Questions were ranked from one (top rank, highest average total score) to 18 (lower rank, lowest average total score).

Second, the “overall score” was entered also using a five-point Likert scale where one represented a low overall priority and five represented a high overall priority. The overall score was also combined by taking a flat average for each question, across all participants. Questions were ranked from one (top rank, highest average overall score) to 18 (lower rank, lowest average overall score).

Third, the “top five” was a simple count of the number of times each research question was listed in the top five priorities of all participants and questions were ranked from one (highest number of counts) to 18 (lowest number of counts).

The results of the ranking exercise were analysed on site, presented and discussed in plenary, and consensus was reached on top priority questions.

## Results

### Policy concerns raised at country-, regional-, and global-level

A total of 26 crosscutting high priority policy issues were identified at country- and regional-level. Nine policy issues were consistently raised in all five regions and related to: i) perception of generic medicines by patients, prescribers and providers, and their preference for branded medicines; ii) knowledge, awareness, and health seeking behaviour of patients; iii) development of central and essential policy documents such as standard treatment guidelines and national essential medicine lists; iv) universal coverage, social health protection and coverage of medicines under health financing arrangements; v) level of public and government funding for health and medicines; vi) inefficiencies in budget disbursement and implementation; vii) medicine pricing policies; viii) capacity of staff and systems for procurement, supply and stock management; and ix) fight against counterfeit medicines. An additional eleven issues were raised in four regions and six issues were raised in three regions. Policy issues raised by less than three regions are considered as low priority and are not presented here.

A total of 23 informants were interviewed at global level, of which six were women. Ten informants originated from a LMIC. Interviewees had expertise either in pharmaceuticals or health systems and worked at headquarters of international multilateral agencies (n = 8), at regional offices of multilateral agencies (n = 4), in a bilateral donor organization (n = 1), in international NGOs (n = 4) and six were researchers in academic institutions. The results of global KIIs were fairly consistent with country- and regional-level results. Global KIIs placed more emphasis on the role of human resources for health in medicine access, although for both groups, the specific policy focus was the impact of financial and non-financial providers’ incentives on access. The second most important domain for global key informants was the same as for countries and regions: sustainable financing and affordable prices. Consistent with data from countries and regions, quality issues came third with global key informants; however, here, a focus on substandard medicines was considered to be more critical than counterfeit medicines as these were seen as a symptom of other system failures outside of quality assurance. In general, all priority policy issues that were identified consistently in all five regions were presented as important concerns for global key informants.

A consolidation of results from country, regional, and global exercises allowed authors to define a final list of 18 crosscutting high-priority policy issues related to ATM in LMICs. Inclusion of priority policy issues in this list was based first on the number of regions where the issue was raised and second according to the number of informants who raised the issue (Table 2). As the results of Global KII were consistent with country- and regional-level results, no additional policy issues raised by Global Key Informants were added to the list of country and regional priorities. A few policy issues among the list of 26 priorities resulting from country- and regional-level exercises were combined as they appeared redundant or strongly linked (e.g., “provider payment methods and financial incentives to providers” was combined with “financial and non-financial incentives to providers”; “procurement and supply of generic medicines” was combined with “quality of generic medicines”).

**Table 2:** Priority policy issues identified by country, region and global stakeholders’ interviews (unranked)

**Priority policy issue**	**No. of regions**	**No. of global KII**
**1. Medicine selection and use**		
Generic medicines: perceptions of patients, communities, prescribers and dispensers of low quality and efficacy of generic medicines. Inadequate demand for branded medicines, perceived as superior to generics.	5	15
Clinical practice guidelines, Standard Treatment Guidelines (STG), National Essential Medicines List (NEML): development, implementation, enforcement, standardization of implementation between public and private sector, procedures for addition and deletion to NEML, STG, formularies, generic policies. Impact of these on medicines use and access.	5	15
Health seeking behaviour of patients, households and communities: knowledge and awareness of general public on medicines, patients’ expectations from health services, adherence to prescribed medication and treatment; self-medication.	5	14
Overuse of medicines: inappropriate use of injections, intravenous perfusions, antibiotics.	3	20
Financial and non-financial incentives for providers: impact of incentives on prescribing practices, quality of care and access. Includes issues related to transparency of incentive systems or the impact of removing financial link between patients and providers.	3	20
**2. Sustainable financing and affordable prices**		
Medicines and health financing arrangements: coverage and reimbursement of medicines under pre-payment and social health insurance schemes, impact on access, out-of-pocket and catastrophic expenditures. Includes resource mobilization for universal coverage of medicines, fragmentation of financing schemes, cost containment policies.	5	18
Resource allocation for health and medicines: government budget for health, funds allocated to health service delivery and medicines. Includes issues related to accountability and disbursement of funds at implementation level, and impact of these on medicine availability and prices.	5	18
Medicine pricing: pricing policies and regulations, and their impact on access, especially mark-up. Includes issues related to transparency, corruption and speculation on medicine prices.	5	18
**3. Leadership and governance**		
Crosscutting policies outside the health sector affecting health and medicines access: such as finance policies or legal issues, coordination and engagement of stakeholders across sectors, and transparency (e.g., regulation and management of conflicts of interest, regulation of incentives and profits above the health sector).	4	14
Governance over the private sector: mapping private sector health service delivery and assessment of training and support needs, governance over the informal pharmaceutical markets, regulation of unethical promotion practices and impact on access.	4	14
Donors’ agenda, funding type and funding mechanisms; impact on access.	3	18
**4. Availability of medicines**		
Procurement, supply and stock management: limited capacity for these functions in resource limited settings, including regulatory capacity and enforcement. Includes issues related to regulation and enforcement of generic policy for procurement and supply.	5	9
Medicine availability in the public sector: comparison with availability in the private sector, consequences on health seeking behaviour, medicine use, price and affordability.	4	9
Geographical accessibility: physical barriers, remoteness, geographical distribution of health services; impact on access.	3	9
**5. Human resources for health**		
Deployment/shortage and training of human resources for health, e.g., in underserved areas; impact on access.	4	20
**6. Quality of medicines and quality assurance systems**		
Counterfeit medicines: regional and national strategies, inspection and border control.	5	NA*
Substandard medicines: technical capacity for quality assurance in LMICs, e.g., laboratory capacity, minilabs; regulation of quality standards and enforcement capacity.	4	16
**7. Medicine information and information systems**		
Monitoring and evaluation systems on rational use, price and quality: data collection, flow of information, adequate and timely use by all stakeholders.	3	5

*Global key informants who mentioned the issue of counterfeit often did mention that counterfeit medicines were symptoms of other problems in LMICs. A few specifically recommended exercising caution and not limiting medicine quality to the issue of counterfeits and some said that the issue of substandard medicines was more important.

### Literature scoping

A total of 1,279 articles were retrieved from PubMed; 619 were found relevant for our study. Results show a distribution of publications predominantly in Sub-Saharan Africa (32.1%), followed closely by the Asian continent (23.6%, with 12.6% in South Asia and 11% in East Asia and the Pacific), and Latin America and the Caribbean (18.6%). A majority of publications focused on the public sector (65.4%) followed by both public and private sector (8.7%); 15.8% of publications studied only the private sector. Half of the publications had a specific urban focus and 12% specifically targeted rural settings.

From reference lists of country and regional priority-setting reports, an additional 72 articles were identified as relevant to the search. Sixteen systematic reviews were also extracted from the review by Gray and Suleman [22]. A total of 707 references were categorized, based on screening through each article, according to the list of priority policy issues in ATM (Table 3).

**Table 3:** Literature scoping results

**Priority policy issue**	**No. of articles**	**Assessing effect**	**Methods**				
			**SR**^**a **^**in LMIC (Total # SR)**	**Quantitative**	**Qualitative**	**Mixed**	**Other**
**1. Medicine selection and use**							
Generic medicines	48	6	1 (1)	8	23	13	3
Clinical practice guidelines, Standard Treatment Guidelines, National Essential Medicines Lists	49	12	4 (4)	8	17	17	3
Health seeking behaviour of patients, households and communities	33	2	1 (3)	2	14	11	3
Overuse of medicines	34	4	2 (2)	11	12	7	2
Financial and non-financial incentives for providers	15	6	1 (4)	0	5	4	2
**2. Sustainable financing and affordable prices**							
Medicine and health financing arrangements	27	12	1 (4)	10	3	10	0
Resource allocation for health and medicines	3	1	0 (0)	1	00	1	1
Medicine pricing	41	12	0 (3)	19	0	9	10
**3. Leadership and governance**							
Cross-cutting policies above the health sector affecting health and medicine access	12	1	1 (1)	0	1	3	7
Governance over the private sector	28	4	1 (2)	4	14	2	6
Donors agenda, funding type and funding mechanisms	14	10	0 (0)	4		3	3
**4. Availability of medicines**							
Procurement, supply and stock management	12	2	0 (0)	5	4	3	0
Medicine availability in the public sector	29	5	1 (2)	6	4	14	3
Geographical accessibility	16	8	1 (1)	5	4	4	2
**5. Human resources for health**							
Deployment/shortage and training of human resources for health	3	2	0 (0)	0	1	2	0
**6. Quality of medicines and quality assurance systems**							
Counterfeit medicines	9	2	3 (3)	1	1	2	2
Substandard medicines	34	9	1 (1)	8	12	5	8
**7. Medicine information and information systems**							
Monitoring and evaluation systems on rational use, price and quality	35	4	1 (1)	4	11	8	11

^a^Systematic reviews.

Priority policy issues for which there are fewer references in the literature include resources allocation for health and medicines; deployment and shortage of human resources for health; counterfeit medicines; procurement, supply and stock management; and crosscutting policies outside the health sector affecting health and medicines. Moreover, some of the priorities are only covered by less robust research methodologies. For example, for the following five priorities, no publication was identified that attempts to assess the effects or outcomes of specific interventions: resource allocation for health and medicines; health seeking behaviour of patients, households and communities; crosscutting policies outside the health sector affecting health and medicines; governance over the private sector and financial and non-financial incentives for providers.

### Formulation of research questions and ranking

Fourteen academic researchers were present during the first half of the meeting, including one private foundation and three researchers who had performed country- and regional-level priority setting exercises. They came from institutions in Brazil, China, Ghana, Iran, Mexico, South Africa, Switzerland, Sweden, Thailand, and the United States of America. Seven stakeholders joined the academic group during the second half of the meeting; they came from international multilateral agencies (n = 2), a government agency (n = 1), NGOs (n = 2), a pharmaceutical company (n = 1), and a private foundation (n = 1). Two WHO officers were also present. Eight participants were female. Research questions addressing the 18 priority policy issues were formulated by the academic researchers, and then reviewed by the second group of stakeholders who introduced minor editorial revisions. While the experts did have the option of developing more than one research question per issue, they jointly decided on a single question corresponding to each policy issue. Table 4 presents the research questions formulated by the group and the results of the ranking exercise according to the three ranking methods. It also disaggregates the results according to the four ranking criteria (Innovation, Impact, Equity, and Lack of Research).

**Table 4:** Results of ranking exercise

**Research questions**	**Total score**	**Overall score**	**Top 5**	**Innovation**	**Impact**	**Equity**	**Lack of research**
1. In risk protection schemes, which innovations and policies improve equitable access to and appropriate use of medicines, sustainability of the insurance system, and financial impact on insurance members?	1	1	1	2	1	2	1
2. What are the impacts of different resource allocation mechanisms in fragmented or decentralized health or pharmaceutical systems on access to and use of medicines?	11	11	14	15	14	8	10
3. What are the impacts of different pricing policies and strategies on medicine prices, availability and use?	6	3	8	10	11	3	3
4. What is the impact of different strategies on perception and use of quality assured low-cost generic medicines by key stakeholders including patients, prescribers, dispensers, regulators?	10	10	10	11	10	10	12
5. What is the impact of individual or combined strategies, in particular regulation and economic incentives, in implementing Standard Treatment Guidelines or Essential Medicines list, on appropriate use of medicines in the public and private sector?	15	14	12	16	15	14	15
6. Which innovative strategies targeting individuals, households, communities and systems improve appropriate demand (health seeking behaviour) for access and use of essential medicines?	4	8	4	3	4	5	6
7. What are the best ways of optimizing supply chain management and improving transparency using a systems perspective to improve access to medicines?	16	17	17	12	16	17	16
8. What is the effectiveness and cost-effectiveness of interventions that can be used to detect and reduce supply and demand of counterfeit (fake/falsified) medicines?	13	15	12	9	13	16	9
9. How do we understand and intervene in labour markets for pharmaceuticals to improve the quality of and access to essential medicines?	12	13	16	14	9	13	7
10. What are effective strategies to reduce substandard medicine production, to improve medicine quality and regularly disseminate results?	14	12	11	12	12	15	12
11. How do non-health sector policies (e.g., industry, trade and intellectual property, legal and constitutional, civil service, transport, banking, education, defence, financial systems, customs) influence access to and use of medicines?	5	6	4	4	4	5	4
12. How do policies and other interventions into private markets (such as information, subsidies, price controls, donation, regulatory mechanisms, promotion practices, etc.) impact on access to and appropriate use of medicines?	2	2	4	1	3	4	2
13. What are the lessons learned from best practices for public sector management of essential medicine programs to improve access and appropriate use of medicines?	17	15	17	18	17	12	17
14. Based on evidence of impact of inappropriate drug use on burden of disease, drug resistance and systems and household expenditures, how can systems most effectively and sustainably scale up interventions?	9	8	8	4	4	9	10
15. What incentives in a health system optimize prescribing, dispensing and sales practices among the full range of providers (public, private, formal and informal)?	8	4	3	4	4	7	7
16. What are the best practices of donor and NGO behaviour in working with stakeholders, to strengthen capacity of national systems to improve access to and appropriate use of medicines?	18	18	14	17	18	18	18
17. Which innovations effectively improve access (including geographic accessibility, social acceptability, availability, and financial accessibility) of under-served communities to essential medicines, including community engagement strategies?	3	7	7	8	8	1	12
18. How can stakeholders use the information available in the system (e.g., price, availability, quality, utilization, registration, procurement) in a transparent way towards improving access and use of medicines?	7	4	2	4	2	10	4

Participants in the ranking exercise were presented with preliminary quantitative analyses of all three ranking methods. They reached a consensus that priority should be given to research questions 1, 12 and 18.

Question 1 (“*In risk protection schemes, which innovations and policies improve equitable access to and appropriate use of medicines, sustainability of the insurance system, and financial impact on insurance members*?”) was overwhelmingly ranked as a top priority, irrespective of the ranking method used. This question also scored as a high priority according to all ranking criteria (top priority with regards to Impact and Lack of Research, second priority with regards to Innovation and Equity). This research topic corresponds to a priority policy issue raised consistently in all five regions and by 18 out of 23 global key informants (Table 2). As confirmed by our literature scoping, medicine publications have only recently shifted towards examining health-financing arrangements and investigating medicine prices in LMICs. Our priority setting exercise confirms an on-going interest in these topics, which are also consistent with the current trend of focussing on universal coverage in a post-Millennium Development Goal world.

The top five ranking method placed Question 18 (*“How can stakeholders use the information available in the system, e.g., price, availability, quality, utilization, registration, and procurement, in a transparent way towards improving access and use of medicines?*”) in second position while the results of the total score and overall score methods pointed to Question 12 (*“How do policies and other interventions into private markets (such as information, subsidies, price controls, donation, regulatory mechanisms, promotion practices, etc.) impact on access to and appropriate use of medicines?*) as a second priority. Question 12 was considered highly innovative and addressing a substantial research gap, as confirmed by our literature scoping. Question 18, related to medicine information, was regarded as potentially achieving high impact.

Consensus was reached among the meeting participants for the above top three ranking research questions that needed attention and investment in the future. Participants suggested that the results of the ranking, disaggregated by ranking criteria, be made publicly available to allow researchers, decision makers and funders to select research questions according to their expertise and their needs, depending on whether they wanted to focus on equity, innovation, impact on health and health systems or on filling existing research gaps.

## Discussion

A list of 18 research questions corresponding to policy issues in ATM raised at country-, regional-, and global-level were thoroughly formulated and ranked during our priority-setting exercise. The prioritized HPSR agenda in ATM corresponds to existing research gaps and sets the tone for future research that is likely to be innovative and to have a higher impact on health, health systems, and equity. The top ranking research questions relate to medicine financing (“*In risk protection schemes, which innovations and policies improve equitable access to and appropriate use of medicines, sustainability of the insurance system, and financial impact on insurance members*?”); medicine information and information systems (*“How can stakeholders use the information available in the system, e.g., price, availability, quality, utilization, registration, and procurement, in a transparent way towards improving access and use of medicines?*”); and to leadership and governance, and more specifically, stewardship over the private sector (*“How do policies and other interventions into private markets (such as information, subsidies, price controls, donation, regulatory mechanisms, promotion practices, etc.) impact on access to and appropriate use of medicines?*). This priority-setting approach has been successful in ensuring that a health systems lens is adopted in ATM research [19]: the top priority research questions are tightly interlinked with three other building blocks of the health system, namely health financing, health information systems and governance [1]. Moreover, all three research questions are related to the building block of service delivery. The other research questions highlight the importance of incentives in the health system, demand-side and equity aspects, and contextual factors outside the health sector.

Viergever et al. have established a checklist of nine common themes of good practice for health research priority setting [23]. Our priority-setting exercise was assessed against this checklist as follows:

1. Context: This priority-setting exercise was the major activity of the inception phase for the ATM project launched by the AHPSR in 2010 (http://www.who.int/alliance-hpsr). The objectives of the project are to increase availability and use of HPSR evidence in the field of ATM, and thereby improve access for the most vulnerable populations in LMICs. Adequate level of funding and human resources were provided by the AHPSR since the start of the project and it was foreseen that a bottom-up exercise would be conducted in select countries to generate context-specific evidence on policy issues and priority research questions.

2. Stepwise comprehensive approach: We adopted a stepwise approach, similar to previous priority-setting exercises conducted by the AHPSR. This is consistent with the approach used by Ranson and Bennett [13], who conclude that more successful approaches for considering HPSR priority-setting are country-driven, interpretive and include a wide range of stakeholders. Consistent with this, the Taskforce on Health Systems Research [10] also puts forward approaches to setting priorities in HPSR, based on consensus views of informed participants.

3. Inclusiveness: Our study starts with a bottom-up, country-driven approach and all prioritised research questions respond to policy issues in ATM raised by countries and regions. We have also paid particular attention to the inclusion of typically neglected, but significant actors such as the pharmaceutical industry.

4. Information gathering: Data collection at country-, regional-, and global-level included KIIs on priority policy issues in ATM as well as a comprehensive literature scoping.

5. Plans to translate priorities into research: From the start, it was known that the AHPSR had available funding for a call for research proposals based on established HPSR agenda (http://www.who.int/alliance-hpsr/alliancehpsr_atmcall_for_eoi.pdf)d.

6. Criteria: In all priority-setting exercises, whether for health interventions, health research, or HPSR, authors insist on the importance of criteria used for ranking [24-29]. The four criteria we used for our priority-setting are considered as relevant in many of the above publications: equity is a common criterion to almost all priority-setting papers above, the expected impact of research on health and health systems is considered by several authors as well as lack of existing and previous research [20,21,24]. Innovation is seldom used by other authors, however, Ranson et al*.* observe that the research questions ranked in their study on health financing were usually not very innovative [20]; introducing innovation as a criteria helps to overcome this specific weakness of previous AHPSR priority-setting exercises. Ranson et al*.*[20,21] concur with COHRED [24] that ranking criteria should be based on consensus among participants and this is especially relevant for HPSR [13]. We have applied this by discussing and adjusting ranking criteria prior to conducting the ranking exercise; stakeholders suggested two alternate ranking methods (overall score and top five list) which were used to discuss the final prioritized agenda.

7. Methods: Viergever et al*.*[23] suggest that priority-setting methods may be consultative-based, metrics-based, or a combination of both, while the Taskforce on Health Systems Research [10] and Ranson and Bennett [13] support the use of qualitative methods to prioritize HPSR agendas. Our method was a mixed approach, combining qualitative data collected at country-, regional-, and global-level, a consensus-based approach to formulating the final research questions, a metrics-based approach to the ranking exercise, and finally, consensus-building towards the final agenda.

8. Transparency: The entire process has been transparent with each step having been documented starting with country- and regional-level reports and ending with a full research report following the ranking workshop.

9. Evaluation: The process has not yet undergone any formal evaluation, but is assessed against the checklist of nine common themes of good practice for health research priority-setting as established by Viegever et al*.*[23].

Particular attention has been devoted to mitigating some of the weaknesses of prior priority-setting exercises [20,21]. The lack of standardized methodology for country- and regional-level exercises is one such limitation. To address this, some level of standardization was introduced by holding an inception meeting prior to field data collection (Cambodia, October 2010). Extensive discussions took place to understand the analytical framework, identify determinants of ATM at local, national and international levels that should guide the data collection process and map stakeholders. However, no further standardization was introduced beyond the conceptual level in order to provide sufficient flexibility for the development of context-specific methodologies, in particular with regard to survey tools. In previous exercises, it was documented that key informants had difficulty formulating research questions, and felt more comfortable discussing policy issues. In response to this limitation, exclusive time has been devoted to formulation of research questions by experienced academic researchers during the consultation and ranking workshop. On the other hand, using the expertise of academic experts gathered in a global meeting to formulate research questions may bias the questions towards global level research priorities; however, the presence of researchers from LMICs in the meeting, especially lead country and regional investigators, mitigated such bias^e^. Another limitation of the formulation of research questions was that an in-depth triangulation of research priorities with existing research could not take place; instead academic experts referred to a scoping of the literature, with limited consideration of quality of published papers. This was combined with their own knowledge of available research in the field.

As in any other exercise of this kind, the choice of the frameworks guides the way in which policy concerns are voiced during KIIs. The WHO framework [18] is largely centred on health sector level issues. Such limitation was mitigated by the stepwise comprehensive and inclusive approach adopted for data collection, especially mapping and inclusion of stakeholders at country-, regional-, and global-level.

An intermediate result of our research consists of 17 country-level priority-setting exercises that can be used by national decision-makers to identify important gaps in their pharmaceutical policies and programmes. Summaries of country- and regional-level results are available on the AHPSR website and country research teams pursue their efforts of disseminating these results in national and international level, including in peer-reviewed publications [30-32]. Further, the proposed prioritized HPSR agenda in ATM comprises 18 priority research questions, of which only three have been selected as top priority for the AHPSR Call for Expressions of Interest. Obviously, this investment is limited. Academic institutions at national and international level as well as funding agencies may use this research agenda to orient their efforts and investments towards policy-relevant research in ATM. Being a step-wise priority-setting process, inclusive of a range of stakeholders, including donors, this exercise serves to support policy-relevant research by demonstrating the need and desire for it across stakeholders and by bringing together those interested in the field, leading to improved consolidation of both research and policy-making efforts. Several agencies present at the Global Stakeholders Meeting have already expressed willingness to support this research agenda.

The authors hope that this paper will contribute to the overall science – both in terms of methodological approaches and findings – of priority setting for investment in health research. It is clear, however, that much remains to be done in strengthening the field of priority-setting. First, many organizations funding health or development research require broader lists of priorities as they may need to allocate research across sectors or broadly within the health sector. A broad, cross-sectoral priority-setting exercise might be particularly relevant as the international community establishes – and identifies strategies for addressing – the post-2015 development goals [33]. Second, in order for national-level policy-makers to decide how to spend scarce research resources, priority-setting exercises may need to be carried out at the country-level. This requires a commitment to both building up the skills for conducting priority-setting exercises, and using their results. Relatively few LMICs regularly conduct priority-setting exercises for health research (the most cited examples being Malaysia and Brazil). Third, priority-setting exercises at global level tend to look at demand for, and supply of, research evidence at a single point in time. In fact, the issues of greatest priority to policy-makers and the corresponding evidence base are fast changing. This calls for priority-setting processes that are dynamic (“real-time”), or at least revised at regular intervals.

## Conclusions

The priority-setting process described in our paper established a HPSR agenda in ATM, with 18 prioritized and thoroughly formulated research questions. The top-priority questions focused on: i) medicine financing within risk protection schemes; ii) medicine information; and iii) interventions into private markets. The agenda adopts a health systems perspective and will guide innovative research likely to bear a higher impact on health, health systems, and equity.

## Endnotes

^a^According to the WHO, “Essential medicines are those that satisfy the priority health care needs of the population. They are selected with due regard to public health relevance, evidence on efficacy and safety, and comparative cost-effectiveness.” [http://www.who.int/topics/essential_medicines/en/] Accessed 3 March 2013.

^b^Summaries of country- and regional-level priority setting exercises can be found at the following link [http://www.who.int/alliance-hpsr/projects/medicines/en/index.html].

^c^This excluded Rwanda and Ghana which started later than other countries and had not submitted their draft report when the global-level interviews were designed.

^d^Accessed 18 January 2013.

^e^It should be noted that lead country and regional investigators did not participate in the ranking exercise.

## Abbreviations

AHPSR: Alliance for Health Policy and Systems Research; ATM: Access to medicines; HPSR: Health Policy and Systems Research; KIIs: Key informant interviews; LMICs: Low- and middle-income countries.

## Competing interests

The authors declare that they have no competing interests.

## Authors’ contributions

MB conceptualized the study; coordinated data collection and data analysis at country- and regional-level; conceived and coordinated data collection at global-level and analysed the results; conceived and facilitated the final stakeholders consensus meeting; analysed ranking results; and wrote the manuscript. DJ contributed to data analysis at country-, regional- and global-level; conceived and conducted the literature scoping; facilitated the final stakeholders’ consensus meeting; analysed ranking results; and contributed to writing the manuscript. JH extracted data from country and regional reports; conducted data collection and data analysis at global-level; and commented and approved the manuscript. RL provided advice on the study design, conceptual framework, data collection at country-, regional- and global-level; and facilitated the final stakeholders’ consensus meeting and analysis of ranking results. He has read and approved the manuscript. KR has contributed to conceiving the consensus building process; facilitated the final stakeholders’ consensus meeting; analysed the ranking results; and contributed to writing the manuscript. The AHPSR network of researchers on ATM conceived and conducted the country and regional-level priority-setting exercises and wrote the corresponding research reports. All authors read and approved the final manuscript.

## Authors’ information

MB is responsible for the Alliance HPSR’s programme of work on Access to Medicines, including primary research in LMICs based on the top priority research questions identified in this study.

Alliance for Health Policy and Systems Research network of researchers on Access to Medicines (in alphabetical order): Daniel Arhinful (Noguchi Memorial Institute for Medical Research, University of Ghana); Samer Jabbour (American University of Beirut, Lebanon); Vera Lucia Luiza (National School of Public Health, Oswaldo Cruz Foundation, Brazil); Chean Men (Centre for Advanced Studies and Chean-Jaco Co, Cambodia); Chuc Nguyen Thi Kim (Hanoï Medical University, Vietnam); Joseph Ntaganira (School of Public Health, National University of Rwanda); Claudine Ntsama Essomba (University of Yaoundé I, Cameroon); Arash Rashidian (Tehran University of Medical Sciences, Iran); Sakhtivel Selvaraj (Public Health Foundation of India); Lamphone Syhakhang (Ministry of Health, Lao PDR) and Shehla Zaidi (Agha Khan University, Pakistan).

## Supplementary Material

Additional file 1 Literature scoping: search strategy. Click here for file

Additional file 2 Databases searched by country, regional and global research teams. Click here for file
