# Supplementary material for: Health policy and systems research in access to medicines: a prioritized agenda for low- and middle-income countries
Source: Health Res Policy Syst. 2013 Oct 14;11:37. doi: 10.1186/1478-4505-11-37 (PMC3854087; doi:10.1186/1478-4505-11-37)
Supplement: Additional file 1 — Literature scoping: search strategy. [file 1478-4505-11-37-S1.pdf]

Appendix 1

Literature scoping: search strategy

Medicines

1. drug[Ti] OR drugs[Ti] OR pharmaceutical[Tiab] OR pharmaceuticals[Tiab] OR medicines[Ti] OR "Prescription Drugs"[Mesh] OR "Nonprescription Drugs"[Mesh] OR "Drugs, Generic"[Mesh] OR "Drugs, Essential"[Mesh] OR "Essential Drugs"[TIAB] OR "Essential Medicines"[TIAB] OR "Essential medicine"[tiab] OR "Counterfeit Drugs"[Mesh] OR "Drugs, Essential"[Substance] OR "Pharmaceutical Preparations"[Substance] OR "substandard medicines"[tiab] OR "substandard drugs"[tiab] OR ARV[ti] OR ARVs[ti] OR antiretrovirals[ti] OR "antiretroviral"[ti] OR "pharmacies"[mesh]
2. "Drug Abuse"[tiab] OR "Drug Possession"[tiab] OR "narcotic abuse"[tiab] OR "narcotic use"[tiab] OR narcotics[tiab] OR "substance abuse"[tiab] OR poison[tiab] OR poisoning[tiab] OR venom[tiab] OR "substance use"[tiab] OR cocaine[tiab] OR heroin[tiab] OR marijauna[tiab] OR pot[tiab] OR "salvia divinorum"[tiab] OR "street drugs"[mesh] OR ("Diagnostic Techniques and Procedures"[Mesh] NOT "Diagnostic Tests, Routine"[Mesh])
3. 1 NOT 2

Medicines Selection and Use

4. "Drug Prescriptions/legislation and jurisprudence"[Mesh] OR "Drug Utilization/legislation and jurisprudence"[Mesh] OR "Drugs, Generic "[Mesh] OR "polypharmacy"[tiab] or "polypharmacies"[tiab] or "poly pharmacy"[tiab] or "poly pharmacies"[tiab] OR "prescription filling"[tiab] OR "Self Medication"[Mesh] OR "Drug Prescriptions"[mesh] OR "rational use"[tiab] OR "public awareness"[tiab] OR "Practice Guidelines as Topic/standards"[Majr]

Sustainable Financing and Affordable Prices

5. "Health Expenditures"[Mesh] OR "Insurance, Health, Reimbursement"[Mesh] OR "Insurance, Pharmaceutical Services"[Mesh] OR "Cost Sharing"[Mesh] OR affordable[Ti] OR affordability[Ti] OR “financial incentives”[tiab] OR “monetary incentives”[tiab] OR "Non-financial incentives"[TIAB] OR "Non-monetary incentives"[TIAB] OR “financial incentive”[tiab] OR “monetary incentive”[tiab] OR "Non-financial incentive"[TIAB] OR "Non-monetary incentive"[TIAB] OR "financing"[TIAB] OR "Financial Management"[Mesh] OR "out of pocket"[tiab] OR "out-of-pocket"[tiab] OR "OOP"[tiab] OR "social health insurance"[tiab] OR "social health protection"[tiab] OR "pricing mechanisms"[tiab] OR "cost containment"[tiab] OR "resource generation"[tiab] OR "Resource Allocation"[Mesh] OR "funding type"[tiab] OR "funding source"[tiab] OR "subsidies"[tiab] OR "subsidy"[tiab] OR "Universal Coverage"[Mesh] OR "Healthcare Disparities"[Mesh] OR ("Drug Costs/legislation and jurisprudence"[Mesh] OR " Economics, Pharmaceutical/legislation and jurisprudence"[Mesh] OR "Fees, Pharmaceutical/legislation and jurisprudence "[Mesh])

Leadership and Governance

6. Transparency[tiab] OR accountability[tiab] OR "regulatory capacity"[tiab] OR "regulatory capacities"[tiab] OR "corruption"[tiab] OR "national policy"[tiab] OR "national policies"[tiab] OR "unethical promotion"[tiab] OR "Advertising as Topic/legislation and jurisprudence"[Mesh] OR "Advertising as Topic/methods"[Mesh]OR "patents as topic"[Mesh] OR "productive partnerships"[tiab] or "productive partnerships"[tiab] OR "informal market"[tiab] OR "informal markets"[tiab] OR "Legislation, Pharmacy"[Mesh]

Medicines Availability

7. "drug supply chain"[tiab] OR "procurement"[tiab] OR "stock management"[tiab] OR "supply management"[tiab]

Human Resources for Health

8. ((("Health Manpower"[Mesh] OR "Health Personnel"[Mesh] OR "health worker"[tiab] OR "health workers"[tiab]) AND (deployment [tiab] OR shortage[tiab] OR "Motivation"[Mesh] OR "Reimbursement, Incentive"[Mesh] OR "training"[tiab] or "capacity building"[tiab] or

"capacity-building"[tiab])) OR "Staff Development"[Mesh] OR "Physician Incentive Plans"[Mesh]) OR "provider payment"[tiab])

Medicines Quality and Quality Assurance Systems

9. "Quality Assurance, Health Care"[Mesh] OR "laboratory capacity"[tiab] OR "Clinical Governance"[Mesh] OR "informal market"[tiab] OR "informal markets"[tiab] OR "Drug Monitoring/adverse effects"[Mesh] OR "pharmacovigilance"[tiab] OR "Counterfeit Drugs"[Mesh] OR "Fake Drugs"[TIAB] OR "Counterfeit Medicines"[TIAB] OR "Drug Counterfeiting"[TIAB] OR "poor quality medicines"[tiab] OR "Adverse Drug Reaction Reporting Systems/legislation and jurisprudence"[Mesh] OR "Adverse Drug Reaction Reporting Systems/standards"[Mesh] OR “substandard”[tiab]

Medicines Information and Information Systems

10. ("Drug Industry/education"[mesh] OR "Drug Information Services/legislation and jurisprudence"[Mesh]) OR “INVENTORY CONTROL”[TIAB]

LMICS

11. ("Developing Countries"[Mesh] OR Africa[Mesh] or "Africa South of the Sahara"[Mesh] or Asia[Mesh] or "South America"[Mesh] or "Central America"[Mesh] OR Africa[tiab] or Asia[tiab] or "South America"[tiab] or "Latin America"[tiab] or "Central America"[tiab]) or ("American Samoa"[tiab] or Argentina[tiab] or Belize[tiab] or Botswana[tiab] or Brazil[tiab] or Bulgaria[tiab] or Chile[tiab] or Comoros[tiab] or Costa Rica[tiab] or Croatia[tiab] or Dominica[tiab] or Equatorial Guinea[tiab] or Gabon[tiab] or Grenada[tiab] or Hungary[tiab] or Kazakhstan[tiab] or Latvia[tiab] or Lebanon[tiab] or Libya[tiab] or Libia[tiab] or Libyan[tiab] or Lithuania[tiab] or Malaysia[tiab] or Mauritius[tiab] or Mexico[tiab] or Micronesia[tiab] or Montenegro[tiab] or Oman[tiab] or Palau[tiab] or Panama[tiab] or Poland[tiab] or Romania[tiab] or Russia[tiab] or Seychelles[tiab] or Slovakia[tiab] or South Africa[tiab] or "Saint Kitts and Nevis"[tiab] or "Saint Lucia"[tiab] or "Saint Vincent and the Grenadines"[tiab] or Turkey[tiab] or Uruguay[tiab] or Venezuela[tiab] or Yugoslavia[tiab] or Mayotte[tiab] or "Northern Mariana Islands"[tiab] or "Russian Federation"[tiab] or Samoa[tiab] or Serbia[tiab] or "Slovak Republic"[tiab] or "St Kitts and Nevis"[tiab] or "St Lucia"[tiab] or "St Vincent and the Grenadines"[tiab])or (Albania[tiab] or Algeria[tiab] or Angola[tiab] or Armenia[tiab] or Azerbaijan[tiab] or Belarus[tiab] or Bhutan[tiab] or Bolivia[tiab] or "Bosnia and Herzegovina"[tiab] or Bosnia[tiab] or Cameroon[tiab] or China[tiab] or Colombia[tiab] or Congo[tiab] or Cuba[tiab] or Djibouti[tiab] or "Dominican Republic"[tiab] or Ecuador[tiab] or Egypt[tiab] or El Salvador[tiab] or Fiji[tiab] or "Georgia (Republic)" [tiab] or Guam[tiab] or Guatemala[tiab] or Guyana[tiab] or Honduras[tiab] or "Indian Ocean Islands"[tiab] or Indonesia[tiab] or Iran[tiab] or Iraq[tiab] or Jamaica[tiab] or Jordan[tiab] or Lesotho[tiab] or "Macedonia" [tiab] or "Marshall Islands"[tiab] or Micronesia[tiab] or "Middle East"[tiab] or Moldova[tiab] or Morocco[tiab] or Namibia[tiab] or Nicaragua[tiab] or Paraguay[tiab] or Peru[tiab] or Philippines[tiab] or Samoa[tiab] or "Sri Lanka"[tiab] or Suriname[tiab] or Swaziland[tiab] or Syria[tiab] or Thailand[tiab] or Tonga[tiab] or Tunisia[tiab] or Turkmenistan[tiab] or Ukraine[tiab] or Vanuatu[tiab] or "Cape Verde"[tiab] or Gaza[tiab] or Georgia[tiab] or Kiribati[tiab] or Macedonia[tiab] or Maldives[tiab] or Palestine[tiab] or "Syrian Arab Republic"[tiab] or "West Bank"[tiab])or (Afghanistan[tiab] or Bangladesh[tiab] or Benin[tiab] or "Burkina Faso"[tiab] or Burundi[tiab] or Cambodia[tiab] or "Central African Republic"[tiab] or Chad[tiab] or Comoros[tiab] or "Democratic Republic of the Congo"[tiab] or "Cote d'Ivoire"[tiab] or Eritrea[tiab] or Ethiopia[tiab] or Gambia[tiab] or Ghana[tiab] or Guinea[tiab] or Guinea-Bissau[tiab] or Haiti[tiab] or India[tiab] or Kenya[tiab] or Korea[tiab] or Kyrgyzstan[tiab] or Laos[tiab] or Liberia[tiab] or Madagascar[tiab] or Malawi[tiab] or Mali[tiab] or Mauritania[tiab] or Melanesia[tiab] or Mongolia[tiab] or Mozambique[tiab] or Myanmar[tiab] or Nepal[tiab] or Niger[tiab] or Nigeria[tiab] or Pakistan[tiab] or "Papua New Guinea"[tiab] or Rwanda[tiab] or Senegal[tiab] or "Sierra Leone"[tiab] or

Somalia[tiab] or Sudan[tiab] or Tajikistan[tiab] or Tanzania[tiab] or East Timor[tiab] or Togo[tiab] or Uganda[tiab] or Uzbekistan[tiab] or Vietnam[tiab] or Yemen[tiab] or Zambia[tiab] or Zimbabwe[tiab] or Burma[tiab] or Congo[tiab] or Kyrgyz[tiab] or Lao[tiab] or "North Korea"[tiab] or "Solomon Islands"[tiab] or "Sao Tome"[tiab] or Timor[tiab] or "Viet Nam"[tiab])or ("developing country"[tiab] OR "developing countries"[tiab] OR "developing nation"[tiab] OR "less developed country"[tiab] OR "less developed countries"[tiab] OR "under developed country"[tiab] OR "under developed countries"[tiab] OR "poor country"[tiab] OR "poor countries"[tiab])or ("middle income country"[tiab] or "middle income countries"[tiab] or "low income country"[tiab] or "low income countries"[tiab]) or (lmic[tiab] or lmics[tiab])

12. (japan[ti] OR taiwan[ti] OR singapore[ti] OR "hong kong"[ti] OR japanese[ti] OR taiwanese[ti] OR "south korea"[ti] OR "south korean"[ti]))

13. 11 not 12

<u>Combinations (all limited to 2003- 2012):</u>

Medicines Selection and Use : (#3 AND #13) AND #4
Sustainable Financing and Affordable Prices: (#3 AND #13) AND # 5
Leadership and Governance: (#3 AND #13) AND #6
Medicines Availability: (#3 AND #13) AND # 7
Human Resources for Health: (#3 AND #13) AND #8
Medicines Quality and Quality Assurance Systems: (#3 AND #13) AND #9
Medicines Information and Information Systems : (#3 AND #13) AND #10
