# Supplementary material for: Health policy and systems research in access to medicines: a prioritized agenda for low- and middle-income countries
Source: Health Res Policy Syst. 2013 Oct 14;11:37. doi: 10.1186/1478-4505-11-37 (PMC3854087; doi:10.1186/1478-4505-11-37)
Supplement: Additional file 2 — Databases searched by country, regional and global research teams. [file 1478-4505-11-37-S2.pdf]

Appendix 2

Databases searched by country, regional and global research teams

African Index Medicus, African Journals On Line, Cochrane Database of Systematic Reviews, Canadian Cochrane Network and Centre Database, CINAHL, Database of Abstracts of Reviews of Effects, Eastern Mediterranean Region Index Medicus, EconLit, EMBASE, ELDIS, Google Scholar, Health Systems Evidence (McMaster University), HINARI, ISI, LILACS, MEDLINE, PubMed, REMED, SCOPUS, Sociological Abstracts, Web of Science, WHOLIS

Farsi language: Iran MEDEX, SID

Databases of international and regional organizations: MENA UNDP, UNICEF, World Bank, WHO EMRO
